# Liquid gold: do we need to fraction fresh colostrum for oral immunotherapy in premature infants?

**DOI:** 10.1186/s13006-022-00471-y

**Published:** 2022-04-13

**Authors:** Anish Pillai, Isha Jhaveri, Sachin Sakharkar, Nandkishor Kabra

**Affiliations:** 1 Department of Neonatology, Surya Hospitals, 101-102, Mangal Ashirwad, S V Road, Santacruz West, Mumbai, Maharashtra 400054 India; 2 Department of Pediatrics, Surya Hospitals, Mumbai, India

Dear Editor,

We read with interest the trial on administration of breast milk cell fractions to premature neonates with birth weight equal to or less than 1800 g by Fallahi et.al [1]. The authors reported a significant decrease in mortality with administration of breast milk fractions to neonate. The current study is novel and adds to the literature around importance of early breast milk in improving outcomes. However, we would like to highlight certain concerns that arose while reading this paper.

Oropharyngeal colostrum administration in preterm infants has shown to increase levels of urinary secretary IgA and lactoferrin in preterm infants [2, 3]. In this study, the centrifuged fraction of breast milk expressed within first 6 h of birth was administered to preterm neonates. Although the method of sampling breastmilk cell fraction has been described in detail, the authors do not mention if any quality check was performed to determine the number and type of cells present and factors that may have been lost during the sampling procedure. It is well known that colostrum expressed within the first hours of life is rich in cells and growth factors [4]. We ponder whether it is worthwhile altering a natural ingredient like colostrum which is unique and designed for each baby; since safety and benefits of oral immunotherapy with colostrum has been well described in trials [5]. Also, the study mentions that a large part of the supernatant was removed with cotton swabs. This practice may not be ethical or feasible, since the primary challenge with oral immunotherapy is lack of availability of mothers’ own milk during first few hours after delivery.

The authors mention blinding was not possible due to the nature of the intervention. However, blinding could have been performed by giving colostrum directly to the control group in the same manner as the intervention. In addition, since oral immunotherapy with colostrum is standard of care in many centers, we ponder if using colostrum as control would have altered the study results. The authors do not mention whether the allocation concealment was done or not, and which method was used to prevent selection bias.

The primary outcome of the study was decrease in the risk of necrotizing enterocolitis (NEC), but the authors do not mention regarding other evidence-based strategies that impact NEC such as probiotics, standardized feeding regime and donor human milk availability in their unit. The baseline incidence of NEC mentioned for sample size calculation was 11%, based on the study by Stoll et al. [6]. However, the reference study comprised of extremely low birthweight infants, whereas the present study had bigger babies with birth weight < 1800 g. The authors found the incidence of NEC to be 7% in both the groups, raising concerns regarding validity of the results. Lastly, the reason for decreased mortality noted in the intervention group is unclear; since none of the major neonatal morbidities such as late onset sepsis, necrotizing enterocolitis, chronic lung disease, growth velocity or duration of hospital stay were different between the groups. Thus, although early colostrum is important for premature infants, it is unclear if processing and fractioning breastmilk would confer any additional advantage in this population.

## Data Availability

Not applicable.
